# Novel Expression of Thymine Dimers in Renal Cell Carcinoma, Demonstrated through Immunohistochemistry

**DOI:** 10.3390/biomedicines10112673

**Published:** 2022-10-23

**Authors:** Dorin Novacescu, Talida Georgiana Cut, Alin Adrian Cumpanas, Felix Bratosin, Raluca Amalia Ceausu, Marius Raica

**Affiliations:** 1 Doctoral School, Victor Babes University of Medicine and Pharmacy Timisoara, E. Murgu Square, Nr. 2, 300041 Timisoara, Romania; 2 Department XIII, Discipline of Infectious Diseases, Victor Babes University of Medicine and Pharmacy Timisoara, E. Murgu Square, Nr. 2, 300041 Timisoara, Romania; 3 Center for Ethics in Human Genetic Identifications, Victor Babes University of Medicine and Pharmacy Timisoara, E. Murgu Square, Nr. 2, 300041 Timisoara, Romania; 4 Department XV, Discipline of Urology, Victor Babes University of Medicine and Pharmacy Timisoara, E. Murgu Square, Nr. 2, 300041 Timisoara, Romania; 5 Methodological and Infectious Diseases Research Center, Victor Babes University of Medicine and Pharmacy Timisoara, E. Murgu Square, Nr. 2, 300041 Timisoara, Romania; 6 Department II, Discipline of Histology, Victor Babes University of Medicine and Pharmacy Timisoara, E. Murgu Square, Nr. 2, 300041 Timisoara, Romania; 7 Angiogenesis Research Center, Victor Babes University of Medicine and Pharmacy Timisoara, E. Murgu Square, Nr. 2, 300041 Timisoara, Romania

**Keywords:** renal cell carcinoma (RCC), tumor adjacent healthy renal tissue, thymine dimers (TD), cyclobutane pyrimidine dimers (CPD), lymphatic dissemination, immunohistochemistry, molecular pathology, carcinogenesis, prognosis, biomarkers

## Abstract

Despite significant developments in renal cell carcinoma (RCC) detection and molecular pathology, mortality has been steadily rising. Advanced RCC remains an incurable disease. Better clinical management tools, i.e., RCC biomarkers, have yet to emerge. Thymine-dimers (TDs) were traditionally considered photo-dependent pre-mutagenic lesions, occurring exclusively during ultra-violet light exposure. Non-oxidative, direct, and preferential byproducts of DNA photochemical reactions, TDs, have recently shown evidence regarding UVR-independent formation. In this study, we investigate, for the first time, TD expression within RCC tumor tissue and tumor-adjacent healthy renal parenchyma using a TD-targeted IHC monoclonal antibody, clone KTM53. Remarkably, out of the 54 RCCs evaluated, 77.8% showed nuclear TD-expression in RCC tumor tissue and 37% in the tumor-adjacent healthy renal parenchyma. A comprehensive report regarding quantitative/qualitative TD-targeted immunostaining was elaborated. Two main distribution models for TD expression within RCC tumor tissue were identified. Statistical analysis showed significant yet moderate correlations regarding TD-positivity in RCC tissue/tumor-adjacent healthy renal parenchyma and TNM stage at diagnosis/lymphatic dissemination, respectively, indicating possible prognostic relevance. We review possible explanations for UVR-independent TD formation and molecular implications regarding RCC carcinogenesis. Further rigorous molecular analysis is required in order to fully comprehend/validate the biological significance of this newly documented TD expression in RCC.

## 1. Introduction

The current definition of the term renal cell carcinoma (RCC) embodies a very diverse group of distinctive tumor phenotypes, which manifest extensive genomic pleomorphism and, unsurprisingly, wide variability in clinical behavior and therapeutic response [1,2]. The plethora of emerging RCC subtypes, characterized by an array of not-yet validated, scarcely defined, relatively non-specific, and much-disputed morphologies, mandate the routine use of ancillary studies in clinical practice.

Beyond evolving definitions, even within the classic morphological three-tier classification, i.e., clear cell (cc)RCC, papillary (*p*)RCC, and chromophobe (ch)RCC, standard microscopic evaluation remains highly subjective. RCC morphologies are notoriously heterogeneous, with very frequent overlap, occurring at least focally. For high-grade and/or dedifferentiated RCCs, morphology alone is useless in offering risk stratification, personalized treatment, or providing a robust tumor definition, for that matter. Accurate diagnosis of metastatic origin, where the differential must include non-renal origins as well, is impossible without at least immunohistochemistry (IHC) [2,3]. Undeniably, the growing use of IHC in everyday clinical practice has greatly improved RCC pathological diagnosis objectivity. Even so, the method still has persisting weaknesses, i.e., clone variations, scarcity of validation studies, specificity issues, and interpretative difficulties, especially for subcellular immunoreactions [3,4].

Notwithstanding the progress made so far regarding RCC diagnostics, pathological definitions, and therapeutic modalities, advanced RCC remains incurable, and RCC-specific mortality rates have been steadily increasing since the 1990s (~1.1%/year) [5,6]. This disparity could be accounted for by the concomitant occurrence of some aggravating, occult, metabolic modifications in RCC molecular pathology, possibly brought about by the diverse and dynamic landscape of carcinogenic environmental factors as a consequence of increased/prolonged exposure [5]. Thus, further investigations into RCC carcinogenesis are urgently needed to better comprehend the intimate mechanisms involved in disease occurrence and progression and to possibly identify adequate biomarkers for RCC screening and risk stratification.

Ultraviolet radiation (UVR), a non-ionizing type of radiation, naturally emitted by the sun, is the most well-documented and almost inescapable environmental factor involved in cellular genomic damage, i.e., PDs are the main DNA alteration encountered in sunlight-exposed, terrestrial surface organisms [7]. Thus, unsurprisingly, UVR exposure is known to be central in cutaneous carcinogenesis (malignant melanoma, basal cell carcinoma, and squamous cell carcinoma [8]). In fact, as seen with multiple cellular lineages, exposure to UVR may determine cellular mutation [9], carcinogenesis [10], or cytotoxicity [11] by inducing, in a waveband-dependent manner, mutagenic non-oxidative DNA photo-lesions [12,13] and/or causing oxidative DNA damage, via UV-induced reactive oxygen species (ROS) [14].

UVR primarily targets nucleic acids, its main cellular chromophore, and initiates specific photochemical reactions, which in turn induce genomic damage, represented by specific intra-strand dimerization cross-link lesions between pyrimidine bases (thymine (T) or cytosine (C) in DNA, as well as uracil (U) or C in RNA), called pyrimidine dimers (PDs) [15], with high mutagenic potential [16]. Essentially, UVR is absorbed by a structural covalent double bond within pyrimidine bases, opening up the bond and freeing up a valence for each of the two carbon atoms involved, allowing reactions with neighboring bases along the nucleotide chain. When neighboring a second UV-modified pyrimidine base, direct covalent bonds form between these molecules. Most commonly, this photochemical reaction results in the following molecular geometry: two new bonds between corresponding carbons in the 5th and 6th positions of the neighboring pyrimidine bases, forming a tight four-carbon cyclobutane ring, i.e., cyclobutane pyrimidine dimers (CPDs) [15,17]. Furthermore, these CPDs most frequently form between two Ts, hence the widespread use of the alternative term “thymine dimers” (TDs). Even so, it is worth keeping in mind that these photo-dependent CPDs can also form between other neighboring pyrimidine bases along UVR-exposed DNA strands, i.e., T=C or C=C. These lesions maintain the same cyclobutane ring but occur to a much lesser extent, as even when using 50% GC-rich DNA, the T=T/C=C dimers ratio remains ≥10:1 after UVR exposure [7].

Moreover, additional PD species exist, occurring in a similar photo-dependent manner to CPDs, yet manifesting fundamentally different molecular geometries, i.e., lacking the characteristic cyclobutane ring. Thus, alternatively, a single covalent bond may form between two neighboring UV-modified bases among a pair of carbon atoms, each from adjacent base rings, in different structural positions, the 6th and 4th, respectively, forming 6–4 pyrimidine-pyrimidone photoproducts (6–4 PPs) [17,18]. In any case, PDs constitute extremely chemically stable molecules, and as they form, they will significantly distort the duplex DNA structure, generating a 30° bend and some degree of unwinding [7].

In human skin, direct sunlight exposure, which is mainly constituted of long-length UVAs (95%) and some residual, more energetic, mid-length UVB (5%), unfiltered by the ozone layer [8], will cause sunburn, direct mutagenic genomic damage to the epidermis, and, eventually, if the damage goes unrepaired, will trigger cutaneous carcinogenesis [18,19]. Metabolically, persistent UV-signature mutations [20] and subsequent alterations in genomic integrity, transcription profiles, and proteomics will, in turn, facilitate the dysregulation of various proto-oncogenes/tumor suppressor genes [21,22], thus compromising cellular viability/functional integrity, ultimately resulting in further mutagenesis, carcinogenesis, and/or cellular demise [23].

These photo-dependent pre-mutagenic lesions are extremely frequent within human skin, with each constitutive cell experiencing up to 100 reactions/second of sunlight exposure. Thankfully, the vast majority of the genomic photo-lesions are rectified immediately after occurring, within seconds, before permanent cellular damage can ensue. Human (skin) cells have the ability to recognize and abolish UVR-induced genomic lesions, utilizing a vast and well-coordinated protein apparatus, to seek out UV-corrupted bases, unwind the regional double-helix DNA, and excise the lesion, with a safety margin of ~30 bases around the detected lesion, in a process called nucleotide excision repair (NER). After excision, the constitutive DNA replication machinery will then fill in the gaps and restore the DNA sequence to its original form [15]. Multiple murine studies have shown a strong negative correlation between the innate ability to repair CPDs via NER and the susceptibility to develop skin cancer [24,25,26]. This idea is further bolstered by in vivo observations in humans with genetically defective/reduced NER capabilities, i.e., xeroderma pigmentosum (XP) patients, which manifest a 10,000-fold increased risk of developing skin cancer under the age of 20, as compared to healthy individuals [27].

Recently, multiple reports have contributed to a shift in perspective regarding CPDs and their photo-dependent nature and pathogenesis [28,29]. Traditionally, it has been axiomatically considered that CPDs are the direct, structural, and biochemical effects of UVR on target nuclear chromophores, occurring within pico (10^−12^) seconds of the absorption of UVR energy by exposed pyrimidine bases, i.e., essentially during UVR exposure. This premise profoundly impacted the conceptualization of further experiments, investigating the effects of UVR on nucleic acids and their biological implications in cellular cultures/tissues, particularly those evaluating DNA repair capabilities [28]. In most investigations, CPD levels would usually be quantified prior to, then immediately after UVR exposure, as well as several hours post-exposure. Even so, some earlier papers have indicated a transient increase in CPD levels hours after active UVR exposure had ceased [30,31,32]. These sorts of findings were widely underreported and generally disregarded as mere artifacts. However, in 2015, a paradigm-shifting turn of events occurred, with the publication of a seminal paper conclusively demonstrating that CPDs continue to form post-irradiation, in the complete absence of continuous UVR exposure, in a mechanistically different manner than their direct UVR-absorption counterparts. These pathogenically distinct, recently described, “delayed” genomic lesions were named “dark CPDs” [29]. Unlike their well-investigated photo-dependent counterparts, dark CPDs still lack a comprehensive evaluation of their biological significance [28].

Thus far, most studies have focused on CPD expression and biological significance in UV-exposed areas (animal/human eye and skin cellularity [20,24,32,33,34,35,36,37], pterygium [38], and cutaneous cancers [39,40,41]), mimicking the apparently singular possible scenario of human CPD-driven carcinogenesis, i.e., organ UV-exposure dependent. Very few investigations have analyzed CPD occurrence and/or carcinogenesis in other inherently non-UV exposed cell origins/tissues. Within the little data available, the mutagenic effect of UV-induced CPDs in the replicative DNA of renal cells has already been demonstrated experimentally, albeit only in an animal model (TC7 clone—African green monkey kidney cell line) [42]. Moreover, in a DNA repair study measuring NER in various human fetal cell cultures (brain, intestine, kidney, liver, and skin), post-UVR and N-ethyl-N-nitrosourea exposure showed that, indeed, there is a considerable difference in NER responses following UVR/biochemical genomic injury amongst the various human fetal organs investigated. Skin cells repaired ~50% of the initial PDs within 8 h post-exposure and 65–86% after 24 h, whereas kidney and liver cells were only able to repair 28% and 32%, respectively, in the same 24 h period, suggesting that human fetal kidney cells are the least equipped to deal with mutagenic PDs [43]. Even so, there are no currently available investigations into PD expression in healthy renal tissue, RCCs, or any other non-cutaneous neoplasm, for that matter.

In an attempt to address these gaps in the scientific knowledge while also taking into account the encouraging paradigm shift regarding PD pathogenesis and possible UV-independency, we designed the current study, which aims to investigate, for the first time, CPD expression patterns in RCCs and tumor-adjacent healthy renal tissue, using CPD-targeted IHC (Anti-Thymine Dimer mAb, clone KTM53), on a consecutive single-center series of RCC specimens.

## 2. Materials and Methods

In this study, we retrospectively evaluate, for the first time, the presence and expression patterns of TDs, as genomic lesions in RCC, using IHC. We obtained adequate Institutional Review Board approval (26/28 September 2018) and proceeded to analyze a recent (2016/2017) consecutive RCC series (90 cases). All of the patients have undergone partial or radical nephrectomy directly, without any neoadjuvant systemic therapy. We collected the available clinical data on all cases, namely: age, sex, TNM stage at diagnosis, and its individual components (pT, cN, and cM).

### 2.1. Tissue Processing, Morphologic Evaluation and Case Selection

The paraffin-embedded RCC tissue specimens were sliced using a microtome. The resulting 3 μm–thick tissue samples were set on glass slides and spread out neatly in distilled water to avoid artifact formation. Excess water was removed, and thermal processing followed, then preliminary deparaffination and finally, automated standard protocol Hematoxylin–Eosin (HE) staining was achieved for all 90 resampled RCC specimens, using the Leica Autostainer XL (Leica Biosystems Newcastle Ltd., Balliol Business Park West, Benton Lane, New Castle Upon Tyne NE 12 EW, UK). The HE tumor sections were morphologically evaluated using a Nikon E600 photon microscope.

Our TD-targeted IHC reaction control specimens were external and comprised multiple in vivo healthy skin samples. Skin is known to characteristically express these genomic lesions, due to constant UV exposure, especially in the basal and para-basal layers of the epidermis. Even so, inclusion in the current study required the mandatory presence of healthy renal tissue in the immediate proximity of the tumor on HE staining. As in the case of RCCs, TD expression in healthy renal tissue has not been investigated so far. Furthermore, as seen in clinical practice, pathologists usually find the most relevant diagnostic information in these transitional areas between healthy and malignant tissue (or low/high-grade areas). The cases which met the inclusion criteria were further classified based on predominant morphological growth pattern (the classic 3-tier system for conventional subtypes: 1—ccRCC, 2—pRCC and 3—chRCC, with 4—sarcomatoid dedifferentiation variants of RCC (svRCC) being reported separately) and main nuclear traits (the WHO/ISUP 2017 grading system: G1—absent/inconspicuous, basophilic, nucleoli, at 400×; G2—visible eosinophilic nucleoli at 400×, but not prominent at 100×; G3—prominent eosinophilic nucleoli at 100× magnification; G4—extreme nuclear pleomorphism, giant neoplastic cells and/or any degree of sarcomatoid/rhabdoid dedifferentiation [1]).

### 2.2. TD-Targeted Immunohistochemistry, Microscopic Evaluation, Data Quantification and Digitalization, Statistical Analysis

The morphological evaluation of the RCC samples that met the inclusion criteria was followed by TD-targeted immunohistochemical staining. After the pre-treatment (20 min with ER2—Bond Epitope Retrieval Solution 2, Leica Biosystems Newcastle Ltd., Newcastle Upon Tyne, UK) and peroxide blocking, the incubation time with the primary antibody was 30 min. The primary antibody used was a monoclonal mouse antihuman thymine dimer clone (Kamiya Biomedical Company, Seattle, WA, clone KTM53, dilution 1:10.000). Visualization was achieved by using the Bond Polymer Refine Detection System. 3,3-diaminobenzidine dihydrochloride was used as a chromogen, and hematoxylin as a counterstain. The entire TD-targeted immunohistochemical procedure was performed with Bond Max autostainer (Leica Biosystems), and the method was additionally validated using an external positive control (human skin), which has a very well-documented expression pattern for TDs. Worth noting is the fact that the chosen TD-targeted antibody, clone KTM53, is designed to react specifically with TDs, the most common type of CPDs, usually resulting from the direct interaction between UVR and consecutive pyrimidine bases, in this case, two thymine molecules, in double/single-stranded DNA, without reacting with the usual minor fraction of 6–4 PPs. Thus, by definition, a positive TD cellular immunoreaction can occur solely in the nucleus. For this reason, the definition of cellular positivity was restricted to nuclear TD immunoreactivity exclusively.

With these premises in mind, TD-targeted IHC-stained slide evaluation, quantification of quantitative, and qualitative expression patterns for TDs, suggestive IHC image captures, and other relevant IHC data collections were performed using a GRUNDIUM OCUS^®^40 microscope (Hermiankatu 6G, 33720 Tampere, Finland). All of the slides included in the study database were scanned and digitalized using the Desk Pannoramic Scanner (3D Histech, Budapest, Hungary), then stored in the Histology Department’s Digital Slides Library Case Center.

For each sample evaluated, multiple nuclear TD immunoreaction parameters were documented, starting with the fundamental distinction of positive cellularity localization, i.e., within RCC tumor tissue vs. in the tumor-adjacent healthy kidney tissue. For RCC tumor tissue staining, a simple “yes or no” expression parameter was attributed (labeled TD+/−), with further nuance being conferred by additional stratification using our newly proposed quantitative (SQ) and qualitative (SI) TD immunoreactivity scores, as well as a cumulative expression score (ST), obtained by the addition of the values of the previous two (ST = SQ + SI). Complete definitions are provided in the following table (Table 1).

Regarding tumors adjacent to healthy renal tissue, a similar simplified “yes or no” expression parameter was attributed (labeled HKE+/−). Further nuance was conferred by the quantification of specific organ localizations for these positive, healthy renal cells, i.e., glomerular expression, tubular expression (HTE), and/or stromal/endothelial expression (HSE), in the same simplified “yes or no” manner.

Once all the target parameters (clinical, histological, and immunohistochemical) were collected, we analyzed the resulting consolidated database by using the SPSS v.27 software (IBM, Chicago, IL, USA) on a Microsoft Windows operating system. We performed the Chi-Square and Fisher’s tests to analyze the proportions between the groups of TD-positive and TD-negative RCC cases. The Student’s *t*-test was performed to determine the mean differences of the normally distributed continuous variables. The Spearman correlation coefficient was calculated to determine whether significant associations exist between the variables that were studied. The significance level required was less than or equal to 0.05.

## 3. Results

In total, we found 54 RCC samples that met the established inclusion criteria for the current study. Clinical, morphological, and immunohistochemical data were collected for all 54 RCC cases included in the current study’s database [44].

### 3.1. Descriptive Analysis

Morphological microscopic evaluation of HE stained specimens resulted in a fundamental histological diagnosis stratification of the evaluated RCC cases within the aforementioned conventional RCC pathological subtypes, in a simplistic manner, based solely on the predominant tumor growth pattern, as follows: 1, ccRCCs = 39 cases; 2, pRCCs = eight cases; 3, chRCCs = four cases; and 4, svRCCs = three cases. Nuclear grading further nuanced RCC tumor biology and risk stratification: G1, 15 cases; G2, 26 cases; G3, nine cases; and G4, four cases.

**Figure 1:**
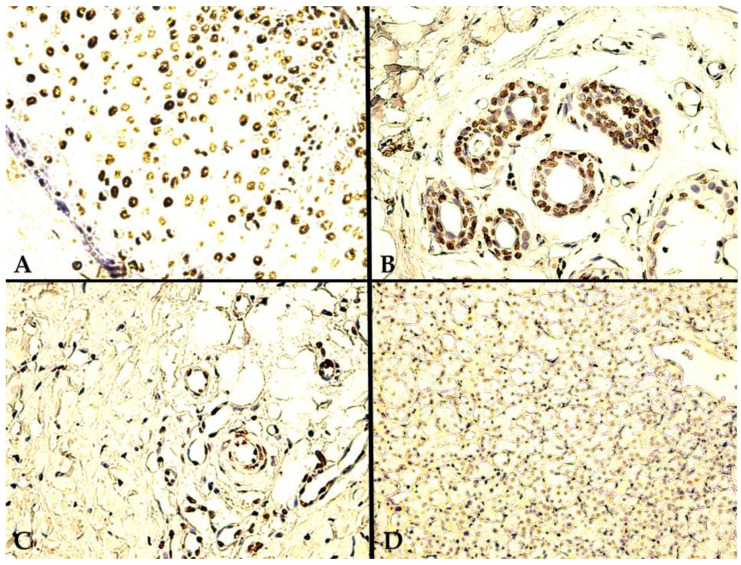
**External positive control/Negative RCC TD-targeted IHC staining**: (**A**) 400×, external positive control, i.e., epidermal TD staining pattern, with an intensely positive nuclear immunoreaction in keratinocytes; (**B**) 400×, positive cutaneous sweat glands; (**C**) 400×, positive cutaneous sweat glands and surrounding stromal endothelial cells; (**D**) 200×, TD-negative ccRCC tumor tissue.

Our TD-target immunoreaction was validated by the external control employed, i.e., multiple in vivo cutaneous tissue samples. These samples were not additionally exposed to UVR, outside of their inherent environmental in vivo exposure, before or after obtaining tissue biopsies. As expected, even though some untreated samples were mostly negative, sporadic areas of diffuse nuclear TD staining were encountered, mainly in basal epidermal cells (Figure 1A), sweat glands (Figure 1B), and stromal endothelial cells (Figure 1C). The final reaction product steadily displayed an intense, exclusively nuclear, brown-colored immunoreaction stain for the aforementioned positive external control cutaneous tissue, with maximum intensity in the epidermis and in the basal and para-basal cellular layers (Figure 1A). In contrast, in Figure 1D, we provide a TD-negative ccRCC landscape with no nuclear immunoreaction in any of the constitutive tumor cellularity. Surprisingly, a pronounced minority within the study population, only 12 (22.2%) tumor tissue TD-negative RCCs were found.

**Figure 2:**
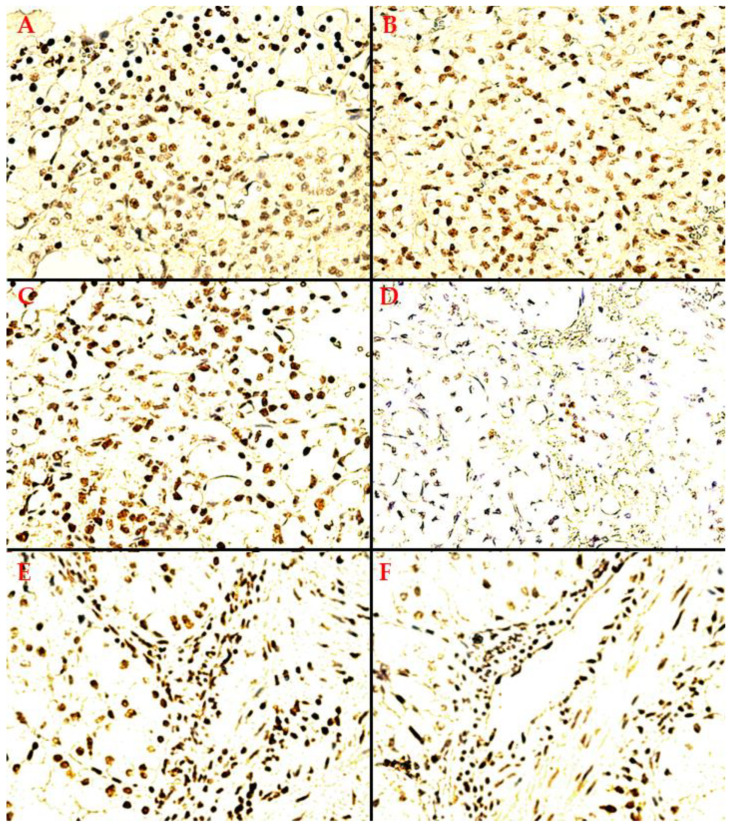
**TD-targeted IHC staining**: (**A**) 400×, model 1 TD immune-reactivity pattern in RCC tumor tissue, heterogeneous distribution within the central tumor areas and along the proliferation front, yet with visible accumulation towards the tumor periphery, ST = +5 (SQ = +3/SI = +2); (**B**) 400×, model 2 distribution, homogenous high density and moderate intensity TD-positive RCC tumor cells, over large areas of tumor tissue; (**C**) 400×, model 2 distribution, homogenous high density and high-intensity positive RCC tumor cells, ST = +6 (SQ = +3/SI = +3) (**D**) 400×, an inconsistent distribution model of low density and low-intensity TD-positive RCC tumor cells, arranged as isolated nests within negative tumor stroma; (**E**) 400×, TD-positive RCC tumor cells, within TD-positive tumor stroma (pleomorphic cellularity); (**F**) 400×, a large TD-positive arterial blood vessel (endothelial cells + parietal myocytes), within the RCC tumor stroma.

Remarkably, out of the 54 samples evaluated, 42 RCCs (77.8%) were positive, manifesting nuclear expression of TDs within RCC tumor tissue. Complete documentation of TD immunoreaction quantification parameters can be found in the following section of Results, namely Statistical Analysis. During the microscopic evaluation of our TD-positive RCCs, we identified two main distribution patterns for TD immunostaining in tumor tissue, namely:

1. A heterogeneous expression pattern, concentrated, in moderate to high density (SQ = +2/+3), at the level of the proliferation front, with variable intensity of TD staining (mostly moderate to high as well, i.e., SI = +2/+3), yet inconsistent in more central tumor areas, or along the transitional area, between tumor and healthy renal tissue (Figure 2A);
2. A homogenous, diffuse, and high-intensity (SI = +2/+3) staining pattern, with the majority of RCC tumor tissue cellularity being positive for TDs (SQ = +2/+3) (Figure 2B,C).

These two predominant, apparently organized, and recurrent patterns of TD nuclear immuno-reactivity amongst the evaluated RCCs were seen, at least partially, in the tumor tissue of a majority (33 samples) of the 42 TD-positive RCC specimens analyzed, having a moderate or high global staining score (ST ≥ 3). An additional, disorganized, seemingly random, low density (SQ = +1), variable intensity (mostly SI = +1/+2), third TD immuno-reactivity pattern distribution model was also encountered (Figure 2D), albeit more rarely. In nine TD-positive RCCs, a weak nuclear TD-stain was encountered, within only a few isolated nests of RCC tumor cells, with mostly negative stromal RCC cellularity, nuclear TD immune-reactivity being distributed in a random manner within the tumor tissue (Figure 2D). Adjacent healthy renal tissue is also generally negative in these 3rd distribution model RCCs, with the exception of two cases, which showed TD expression in both tumor tissue and adjacent healthy tissue (tubular cells). These cases were two females of similar ages, with very different tumor biology, namely: 56 years old, stage 1 ccRCC, G2, pT1b cN0M0, ST = +2(SQ = +1/SI = +1), as opposed to 58 years old, stage 4 svRCC, G4, pT2b cN1M1 (lymph node involvement confirmed through retroperitoneal lymphadenectomy), ST = +4(SQ = +1/SI = +3).

In Figure 2A, the first heterogeneous, nuclear TD immunostaining distribution pattern, seen in the positive RCC tumor tissue samples, is exemplified. This particular RCC sample demonstrates a total TD staining score of ST = +5, with extensive TD positive RCC cellularity (SQ = +3) of moderate intensity (SI = +2), mainly towards the tumor periphery. This TD expression pattern is not maintained in the more central tumor areas, nor consistently along the proliferation front, for that matter. Dominant TD-positive RCC cellularity, with moderate staining, can be seen in the more mitotically active proliferation front. Yet, when approaching the actual transitional area between tumor tissue and adjacent healthy renal tissue, two distribution models have been observed. Either TD immunoreaction may extend into healthy renal tubules in the proximity of the tumor, or tumor-adjacent healthy tissue loses TD expression altogether. In contrast, we included suggestive landscapes for the homogenous TD staining distribution model in TD-positive tumor tissue (Figure 2B,C). The sample in Figure 2B, a high-density (SQ = +3) and moderate-intensity (SI = +2) variant, shows consistent RCC cell positivity. Figure 2C offers a perspective on maximal TD immuno-reactivity seen within this RCC sample cohort, i.e., ST = +6, SQ = +3, SI = +3. Conversely, in Figure 2D, we demonstrate the minimal TD immuno-reactivity encountered (ST = +6, SQ = +3, SI = +3), with a random and inconsistent distribution pattern in RCC tumor tissue.

Regarding RCC tumor tissue TD-positive stroma, there were multiple cell lines that demonstrated nuclear TD-positivity. Usually, TD-positive tumor stroma was encountered in the homogenous tumor tissue TD-staining distribution subgroup and favored the tumor-healthy tissue transitional areas, especially when demonstrating abundant inflammatory infiltrate on HE at this level. In Figure 2E, TD-positive stromal cells (fibroblasts) and diffusely reactive immune cells, mainly macrophages based on morphology, can be seen bordering TD-positive RCC cells. Interestingly, in Figure 2F, an intra-tumoral arterial blood vessel can be seen manifesting TD-positivity, both at the level of the endothelium, both also in constitutive parietal myocytes.

**Figure 3:**
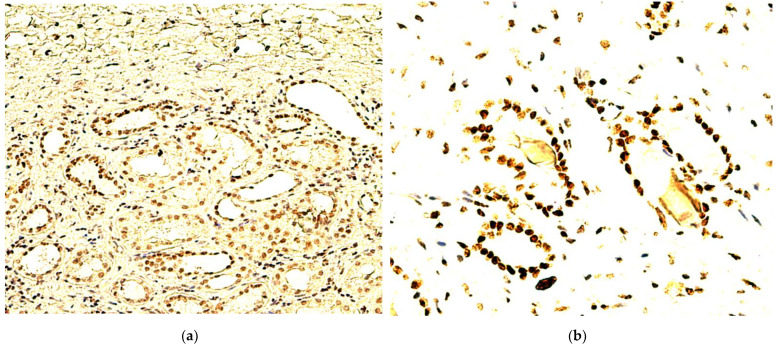
**Tumor-adjacent healthy renal tissue TD-staining patterns.** (**a**) 200×, large number of proximal and distal tubular structures, showing weak to moderate nuclear TD-positivity, bordering TD-negative RCC tumor tissue; (**b**) 400×, high-intensity tubular staining.

Furthermore, we identified a total of 20 RCC specimens (37%) out of the 54 evaluated, which demonstrated TD-positive cellularity in tumor-adjacent healthy renal tissue, yet two of these cases did not manifest TD-positive RCC tumor cellularity (Figure 3a). Out of these two RCCs, manifesting solely tumor-adjacent renal tissue positivity, one was included in our previous WT1 IHC analysis [45], a 51-year-old male patient with an aggressive and systemically disseminated RCC (svRCC histology, G4, stage 3, pT3a cN1M0), and was negative for WT1 in RCC tumor tissue as well. Conversely, the remaining RCC case, showing tubular TD-positivity solely in tumor-adjacent healthy renal tissue, occurred in an older male patient, a 79-year-old, and had profoundly different, almost opposite, RCC characteristics, being a localized, well-differentiated ccRCC, G2, stage 1 (pT1a cN0M0).

Regarding specific TD-positive cellularity and tumor-adjacent renal tissue positivity distribution patterns, no glomerular expression was encountered in any of the 20 positive cases. For all samples, in the healthy renal parenchyma, TD-positive cells were distributed within tubular structures (Figure 2F), especially small, proximal renal tubules, although four cases did manifest TD-positivity solely in the larger, distal collecting ducts. TD tubular immunoreactions showed variable degrees of intensity, ranging from weak to high, as exemplified in Figure 3a,b. The sole exception was seen in a 71-year-old female patient with an advanced ccRCC, G3, stage 3 (pT3a cN1M0), which showed maximal RCC tumor tissue TD-positivity (SQ = +3, SI = +3, ST = +6), with moderate TD expression in proximal renal tubules and, additionally, TD-positive stromal endothelial cells. This was the only case evaluated that showed stromal endothelial TD-positivity in healthy renal tissue, contrasting with the consistent stromal endothelial cell TD-positivity seen in the external positive control healthy skin samples. It seems that this constitutive cutaneous cellular expression pattern is not superimposable with the evaluated healthy renal tissue areas.

### 3.2. Statistical Data Analysis

A total of 54 RCC specimens were eligible for inclusion in the current TD IHC study after the paraffin-embedded specimens were resampled.

The analysis of these samples, obtained through partial or radical nephrectomy from each patient, revealed a total of 42 (77.8%) TD-positive RCC tumor tissue samples and 12 negatives. We ran Chi-Square and Fisher’s tests to analyze the proportions between these two subgroups, TD positive and TD negative RCCs. The majority of patients were men (64.8%), without significant differences between the groups of positive and negative TD samples. The average age was 65.7 years in the TD positive group and 67.5 years in the negative group (*p*-value = 0.499). The most common histologic type of renal cell carcinoma was the clear cell type, which was found in 30 out of 42 patients in the TD positive group, respectively, and nine out of twelve patients in the TD negative group. All of the relevant clinical parameters have been collected and analyzed, namely definitive pathological RCC diagnosis, clinical loco-regional extension and systemic dissemination, cellular aggressiveness, and overall stage at diagnosis, and, as presented in Table 2, no statistically significant differences between study groups were observed. The most frequent stages of diagnosis in the research population were stages 1 and 2 (85.7% within the TD positive group vs. 66.7% within the TD negative group, *p*-value = 0.055).

Table 3 shows a complete description of the previously defined TD immunoreaction quantification parameters for the identified tumor tissue TD-positive RCC cases, i.e., quantitative (SQ), qualitative (staining intensity) (SI), and overall (ST) expression scores for nuclear TD-immunoreactions. The majority of tumor tissue TD-positive RCCs, 18 samples (42.8%), had a moderate value attributed to the quantitative evaluation (SQ = +2), while the most prevalent staining intensity score value, attributed to 19 samples (45.2%) within the qualitative evaluation, was also moderate (SI = +2). This means that, for most cases of tumor tissue TD-positive RCCs, the TD nuclear immunoreaction occurred in 11% to 25% of the total tumor cell population, with a staining intensity marginally less or similar to the external positive control. The overall expression score (ST) ranges in value from +2 to +6, with a high prevalence for the mid-range values: 3 (26.2%), 4 (28.6%), and 5 (23.8%) scores.

**Figure 4:**
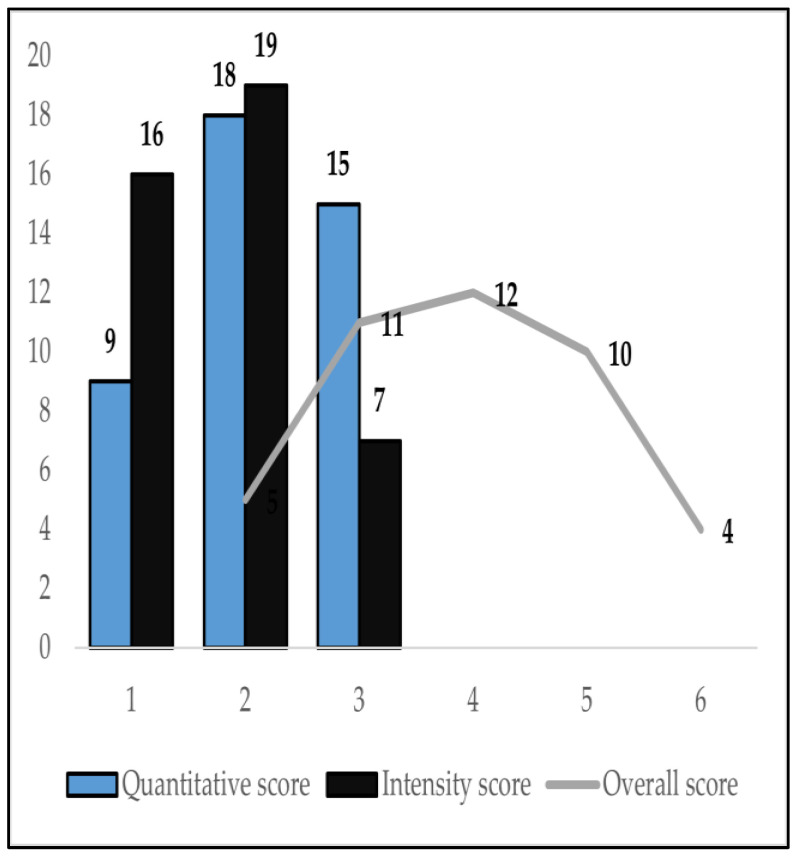
Distribution of TD-staining quantification score values, in tumor tissue TD-positive RCCs.

Indeed, as graphically described in Figure 4, the overall TD expression score (ST), as defined within this analysis, i.e., the sum of the quantitative and qualitative TD-immunoreaction evaluation score values (ST = SQ + SI), offers a much subtler differentiation between tumor tissue TD-positive RCCs, across the board, but especially within the mid-range values. The redistribution of cases, from within the one-sided, individual three-tier individual quantification scores, to a more nuanced five-tier integrative global ranking system, would intuitively seem useful in future tumor tissue TD-expression centered, RCC risk stratification initiatives. However, as reported by the correlation matrix in Table 4, further variable correlation analysis using Spearman’s correlation demonstrated that, in fact, the more simplistic, -“yes or no”- parameter (TD+/−), quantifying the presence of TDs in RCC tumor tissue, manifests a statistically significant (*p*-value = 0.009), moderate negative association (Spearman’s rho = −0.353) with RCC stage at diagnosis, whereas the other, more sophisticated tumor tissue TD-expression quantification scores (SQ, SI, ST), manifest no statistically significant correlations with any of the well-known prognostic clinical parameters for RCC, including in the analysis. Thus, apparently, TD expression is associated with lower RCC stage at diagnosis, yet this correlation needs further validation, as the current study has a relatively small RCC sample cohort, of which almost 80% have a low TNM stage at diagnosis (1 or 2).

Furthermore, the correlation analysis presented in Table 4 also shows a statistically significant moderate negative association between renal cancer subtype and patient’s age (Spearman’s rho = −0.420, *p*-value = 0.002), which is likely due to the small sample size and the simplified, strictly morphological classification, used for RCC subtyping. Unsurprisingly, multiple statistically significant positive correlations amongst the well-established and extensively validated, yet somewhat interdependent, RCC clinical prognosis parameters, which were included in this analysis, namely: Fuhrman grade and TNM stage (Spearman’s rho = 0.295, *p*-value = 0.031); cN and RCC subtype (Spearman’s rho = 0.420, *p*-value = 0.002); cN and Fuhrman grade (Spearman’s rho = 0.356, *p*-value = 0.008); and, obviously, cN and TNM stage (Spearman’s rho = 0.615, *p*-value < 0.001).

Regarding TD-targeted immunostaining parameters within RCC tumor tissue, i.e., TD+/−, SQ, SI, and ST, multiple statistically significant, strong positive correlations have been identified between all of the possible configuration pairs, as these parameters are, obviously, interdependent variables. Therefore, these results are to be expected and have no specific relevance other than that confirming the validity of our statistical analysis method. Interestingly, the simple -“yes/no”- parameter for tumor adjacent healthy renal tissue TD-expression quantification (HKE), manifests statistically significant, moderate positive correlations, with all the individual RCC tumor tissue TD-targeted immunostaining parameters, i.e., SQ (Spearman’s rho = 0.341, *p*-value = 0.012), SI (Spearman’s rho = 0.353, *p*-value = 0.009), and ST (Spearman’s rho = 0.394, *p*-value = 0.003), with the exception of its corresponding “yes/no” variable for tumor tissue TD-expression, TD+/−. Furthermore, when analyzing the only relevant quantification variable of specific intra-organ localizations for these positive, healthy renal cells, namely the healthy renal tissue stromal/endothelial TD expression (HSE) variable, the previously described correlations between HKE and tumor tissue TD expression variables, are no longer encountered for HSE. Surprisingly, the only statistically significant association identified for HSE is with cN, namely a moderate positive correlation between these two variables (Spearman’s rho = 0.356, *p*-value = 0.008), meaning that stromal/endothelial TD expression in tumor-adjacent renal tissue may be a predictive marker for lymphatic dissemination in RCC. This statement obviously represents wishful thinking. Firstly, correlation does not equate to causation, and secondly, the statistical analysis was conducted on a small subset of cases, of which only one case showed positive HSE, albeit a set case had pathological confirmation of retroperitoneal lymphatic dissemination.

## 4. Discussion

Over the past few decades, despite significant improvements, both in imaging technologies for RCC detection, but also in scientific understanding of RCC molecular pathology and tumor biology, RCC mortality rates have been steadily rising, and advanced RCC remains a rapidly progressing, incurable disease. Thus, further investigations are urgently needed in order to develop better clinical tools for RCC diagnosis, risk stratification, and therapeutic response assessment. Hopefully, long-awaited RCC biomarkers that are able to address these clinical needs will soon be developed.

In the current study, we investigate, for the first time, the expression of TDs in RCCs and tumor-adjacent healthy renal tissue using IHC. Remarkably, our results show that a significant majority of evaluated RCC specimens demonstrated TD nuclear expression, both in the actual RCC tumor tissue (77.8%) and also, albeit to a lesser extent, in the surrounding healthy renal parenchyma (37%). These findings are quite controversial as they contradict the well-established pathogenic paradigm of TDs being photo-dependent genomic lesions and occurring exclusively during UVR exposure as non-oxidative byproducts of direct photochemical interactions between UVR and nucleic acids. In these RCC specimens, UVR exposure cannot account for the detected TDs, implying that a different, not yet known pathogenic biochemical mechanism for TD formation must be involved.

In fact, the fundamental conviction that TD induction is photo-dependent may have inadvertently caused a pervasive study design bias. To date, the expression and biological significance of TDs has been almost exclusively investigated in cells/tissues naturally exposed to UVR in the form of direct sunlight, i.e., cutaneous/ocular. Thus, even though TDs have been established as being heavily involved in cutaneous carcinogenesis, their expression and biological roles in other epithelial tumors remain virtually unexplored. To the best of our knowledge, the current IHC investigation of TD expression patterns in RCCs and tumor-adjacent healthy renal tissue is the first of its kind. Thus, our results are novel and have no adequate term of comparison in the existing literature while also lacking validation.

Similarly, with TDs being viewed as occurring exclusively during UVR exposure, a certain degree of reporting bias may have affected earlier TD photo-induction and DNA repair experiments. Thus, although additional increases in TD expression levels were encountered long after UVR exposure had ceased, within multiple in vivo and in vitro TD photo-induction experiments, these findings were generally dismissed as mere methodological artifacts [28]. For example, in a hairless albino murine model, within a transcriptionally inactive gene sequence, there were more TDs found at 2–4 hours post UVB skin exposure than immediately after [30]. In vitro, unstimulated human lymphocytes demonstrated maximal TD levels at 4 hours post-UVC irradiation using IHC. Even though no active NER was documented within 20 h post-exposure, these findings were attributed to improved TD antibody access to target epitopes after the initialization of DNA repair and additional exposure [31]. In vivo, a study using ^32^P-post labeling and high-performance liquid chromatography to evaluate human skin after solar-like UVR exposure (i.e., a two-fold minimal erythema-inducing dose) found that TD(T = C) expression reached peak levels post-exposure, within a 2 h interval [32]. Therefore, even within the existing reports, regarding TD expression patterns, inherent conceptual limitations preclude adequate assessment.

Overall, the current IHC investigation achieved its main goal of detecting and adequately documenting the expression of TDs in RCC tumor tissue and tumor-adjacent healthy renal tissue using a TD-target monoclonal antibody (clone KTM53). Even so, the current study only represents a modest inauguration of a seemingly promising research topic, namely the biological significance of TDs in RCC carcinogenesis, progression, and dissemination. In-depth molecular studies are urgently needed in order to fully comprehend and validate the implications of these novel RCC mutagenic DNA lesions.

### 4.1. Speculative Models and Future Research Perspectives

We hope that our current results, regarding the newly detected expression of TDs in RCC tumors and adjacent healthy renal tissue, albeit isolated and lacking validation, will invigorate further, much-needed research initiatives and will ultimately serve to nuance existing knowledge regarding RCC molecular pathology, UVR-independent CPD formation, and its significance within RCC carcinogenesis and prognosis. Hopefully, this study will represent a stepping stone to adequate RCC biomarker identification and improved RCC clinical management.

Confoundingly, despite extensive and ongoing analysis, even within cutaneous carcinogenesis (particularly for malignant melanomas), the role of TDs remains unclear, with lingering unresolved inconsistencies amongst available reports. Melanomas almost always harbor specific UV-signature mutations, i.e., substitutions between C and T, in areas where pyrimidine bases neighbor each other [46,47]. These UV-signature mutations occur specifically due to amounting unrepaired non-oxidative PDs, causing mechanical distortions of the DNA helical structure [48,49]. However, the UVR-induction of PD formation is wavelength dependent, meaning that exposure to different wavelengths within the UVR electromagnetic spectrum will produce different DNA-damage yields. Thus, short-length UVCs (190–280 nm) produce strictly non-oxidative PDs, while mid-length UVBs (280–320 nm) and, even more so, long-length UVAs (320–400 nm) generate a mixture of non-oxidative (PDs) and oxidative (oxidized bases) genomic lesions [7]. The sunlight reaching the Earth’s surface is comprised of mainly low-energy, long-wavelength UVAs (95%) and a small fraction of UVBs (5%) [18], yet UVBs are much more efficiently absorbed by cellular chromophores, with PDs forming almost instantaneously, upon exposure [50]. Conversely, despite causing melanoma in murine model exposure experiments [29], UVAs are less energetic and incapable of producing the cytosine-containing CPDs that lead to the characteristic C to T substitution, UV-signature mutations, frequently seen in human melanomas [51,52]. Even so, UVAs penetrate the skin deeper and can be absorbed by other secondary cellular chromophores, inducing oxidative changes, and causing indirect DNA damage [18].

Recently, in an effort to address these discrepancies within UV-induced melanoma pathogenesis, a paradigm-shifting seminal paper was published. Herein, the continued formation of CPDs in UVA/UVB-exposed melanocytes, for >3 h after irradiation had ceased, is demonstrated. Additionally, an unusual, alternative biochemical pathway for UV-induced melanoma carcinogenesis is postulated, which solves previous disputes by incriminating melanin as an active participant in CPD formation [29]. The newly defined “dark CPDs”, the major constituent of melanocyte UVA-induced CPDs within this experiment, also demonstrated a cytosine-containing fraction, not seen in CPDs formed during direct UVR exposure, which may account for the aforementioned UV-signature mutations documented in vivo, for human melanomas.

Pathogenically, dark CPDs form in a UVR-independent manner due to the combined effect of UV-induced reactive oxygen and nitrogen species, which will excite an electron in melanin molecules and create a quantum triplet state, holding the energy of a photon, only to transfer set energy to adjacent pyrimidine bases and create CPDs [29]. Thus, via the process of chemiexcitation, i.e., reactions that produce molecules that harbor excited electrons [53], additional dark CPD lesions are latently generated, hours post-UVR-exposure, in a distinctive, oxidative stress-related manner.

To date, very little data are available regarding UVR-independent CPD formation in general, much less in specific cells and tissues (RCC vs. healthy kidney). So far, no definitive biochemical model has been elaborated explaining “dark CPD” occurrence and pathogenesis other than the melanin chemiexcitation proposal. Intuitively, a similar chemiexcitation-based biochemical model may be involved in the UVR-exposure-independent formation of CPDs within other epithelial tissues, i.e., renal/urinary. Obviously, a different renal/urinary tissue-specific protein must act as an energy vector. Additionally, the initial trigger and specific source of superoxide and nitric oxide production remain unidentified.

Notwithstanding the uncertain biochemical mechanism allowing for renal CPD formation, it has already been demonstrated in vitro that unrepaired UV-induced CPDs manifest mutagenic effects in monkey renal cell cultures [42]. Furthermore, human fetal kidney cells have been shown to have a significantly reduced ability to repair CPDs through NER, as compared to other cellular origins from various organs in the same developmental stage [43]. All of the factors combined lend a great deal of plausibility to the idea that CPDs may be actively involved in RCC carcinogenesis, either through tumor suppressor inactivating/proto-oncogene activating mutations [21,22] or cytotoxic effects on tumor-targeting immune response cellularity [11].

In general, as a constitutive part of any tumor biology, an immuno-modulatory imbalance within the tumor microenvironment (TME) is inferred, namely a disparity between antitumor responses and tumor-promoting inflammation [54]. Specifically, regarding RCCs, which are known to illicit particularly intense immune responses and extensive neo-angiogenesis, TMEs are usually extremely pleomorphic and wildly dynamic. Cellularity is mainly comprised of bioactive fibroblasts, adipocytes, neuroendocrine, immune/inflammatory, and endothelial cells dispersed within an equally active extracellular matrix [55]. Thus, RCC cells will implicitly interact with this mixt, stromal, and immune response, cellularity, both directly, via secretion of modulatory, autocrine, and paracrine-acting mediators, as well as indirectly, via proliferative hypoxic/necrotic metabolic events. Precisely this elaborate and well-orchestrated, albeit extremely disruptive, interplay will, in turn, ceaselessly reshape and metabolically redefine RCC biology. The resulting RCC TME heterogeneity is already being carefully investigated and will probably prove to be edifying in the struggle to fully understand RCC carcinogenesis and the plethora of mechanisms involved in progression and therapy resistance [54].

Within the currently evaluated RCC specimens, we mainly found stromal TD-positivity in homogenously, densely, and intensely TD immuno-reactive tumor tissue samples, preferentially at the tumor-healthy tissue interface and especially when demonstrating extensive inflammatory infiltration at this level. Most of the aforementioned biologically active TME cellularity (fibroblasts, endothelial cells, arterial parietal myocytes, and stromal macrophages) has demonstrated nuclear TD immuno-reactivity in the current IHC RCC investigation. The significance of this expression pattern is yet to be determined, but it seems highly likely that these pre-mutagenic lesions have a still unknown, yet apparently important, pathogenic role in RCC carcinogenesis, proliferation, and dissemination, by disrupting TME antitumor cytotoxic responses [11]. As seen within the statistical analysis provided, notwithstanding our obvious study limitations regarding cohort size and simplified, strictly morphological, RCC subtyping procedure, lymphatic dissemination is seemingly positively associated with tumor-adjacent healthy renal tissue TD stromal/endothelial positivity. Much larger cohorts must be evaluated for truly relevant statistical results, which will hopefully validate our initial findings later.

In any case, the modulation of key inflammatory pathways, i.e., Von Hippel–Lindau (VHL), mechanistic target of rapamycin (mTOR), tumor necrosis factor (TNF), and STAT [56], using targeted systemic therapies, represents the cornerstone of contemporary oncological management in advanced RCCs. Following the same principle, there are multiple extensively investigated and readily available molecules (α-tocopherol (vitamin E) [57], isoflavone genistein [58], resveratrol [36]), which have already demonstrated antioxidant and inhibitory properties regarding PD formation. Provided adequate molecular characterization and validation of the novel oxidative/TD-induced RCC carcinogenesis model proposed within the current paper, these molecules may lend themselves to RCC prevention and/or treatment.

In hindsight, before the advent of more targeted therapies (anti-angiogenic tyrosine kinase inhibitors, immune-checkpoint inhibitors), the systemic treatment of metastatic RCCs relied heavily on immunomodulatory cytokines, mainly Interferon (IFN) 1α. This cytokine has proven to be efficient in metastatic RCC therapeutic management, with a 15% overall response rate [59]. These therapeutic results derive from IFN1α’s anti-proliferative and anti-angiogenic effects within RCC tissue, as well as its modulatory cellular differentiation activity via growth factor cross-talk and transcriptional control over key cellular differentiation genes [59,60]. Interestingly, in a very recent murine model investigation, IFN 1α/β demonstrated a novel genomic photo-damage repair activity via NER gene induction and a protective effect against UVR-induced immuno-suppression [61]. Taking into account the findings of our current study, it may be the case that TDs have constituted an occult molecular therapeutic target in metastatic RCCs undergoing immunomodulatory cytokine treatment via IFN 1α-induced improvements in NER capabilities in TD-positive renal/RCC tissue.

Alternatively, beyond TD-induced immunosuppression, a more fundamental RCC carcinogenesis and progression model can be envisioned, resulting from persistent, unrepaired TDs, leading to direct TD-induced mutagenesis and subsequently activating proto-oncogenes and/or inactivating tumor suppressors. Genomic sequencing initiatives, such as The Cancer Genome Atlas (TCGA), have greatly accelerated the process of obtaining accurate genomic mapping for most cancers. So far, full RCC genome characterization has been achieved [62], and pathognomonic RCC signature mutations have been identified, mainly involving the *VHL* (52%) and *PBRM1* (33%) genes [63]. Significantly mutated gene (SMG) clusters for each conventional RCC subtype have also been defined, with RCC signature mutations (in *VHL* and *PBRM1*), alongside other non-specific mutations in *SETD2*, *KDM5C*, *PTEN*, *BAP1*, *MTOR,* and *TP53* [64], being the most relevant in predicting RCC outcomes. In fact, a comparative evaluation of RCC subtypes reported that, among the aforementioned SMGs, only *TP53* and *PTEN* were seen in all conventional subtypes (ccRCC, pRCC, and chRCC), yet they maintained differential, RCC subtype-specific, prognostic value, i.e., *TP53* mutation associated reduced survival rates for ccRCCs and pRCCs, whereas *PTEN* mutation only associated reduced survival rates for chRCCs [62].

Furthermore, being a well-known tumor suppressor gene, normal wild-type *TP53* transcriptional activity is essential for initiating adequate cellular defense responses against genomic injury. *TP53* holds a central role in DNA stability maintenance via the transcriptional induction of p53 transcript-regulated genes involved in p53-dependent DNA repair mechanisms (mainly NER and base excision repair, as shown for UVR-induced, oxidative and non-oxidative DNA lesions, respectively [65,66]). Additionally, the normal p53 transcript sequence is also responsible for the modulation of cellular proliferation, with an inhibitory effect on neo-angiogenesis and multiple functions within the initiation of cell cycle arrest and cellular apoptosis [67,68]. Thus, taking into account the broad array of fundamental anti-proliferative mechanisms dependent on adequate *TP53* transcription, the mutational inactivation of *TP53* functionality will have a powerful carcinogenic effect by incapacitating DNA repair mechanisms and cellular apoptotic functions. Unsurprisingly, a plethora of mutant *TP53*-driven carcinogenesis models have been reported for various tumors of different cellular origins (mammary [69], gastric [70], colorectal [71], cervical [72], ovarian [63], oral [73], and hematological [74]), seeing as over 50% of all human tumors harbor a mutation within the *TP53* gene [75,76].

In the case of RCCs, a recent meta-analysis confirmed that *TP53*-mutant tumors, manifesting the characteristic, subsequent, defective p53 transcript immunohistochemical overexpression, will also demonstrate more aggressive clinical and pathological tumor traits and will hold a much more unfavorable prognosis [77]. Interestingly, the *TP53* gene functionality is quite vulnerable, with evidence that even an isolated, singular missense mutation in the *TP53* transcriptional sequence will be enough to disrupt p53 transcript molecular stability and functionality [78]. Conversely, within UVR-induced murine cutaneous tumors, the *TP53* genomic sequence has demonstrated an inherent propensity and sensitivity for CPD formation within a mutational hotspot located on codon 270, which corresponds to codon 278 in humans [79,80]. Notably, in similar murine cutaneous tumor models, it has been additionally reported that, within *TP53*’s codon 270, DNA repair through NER is significantly less active in comparison to neighboring codons within the *TP53* gene sequence, allowing for a more pronounced CPD accumulation at this level and, implicitly, a higher rate of CPD-induced mutations [81]. Taking into account the aforementioned existing evidence regarding *TP53* genomic vulnerability, coupled with the newly reported, significant TD expression patterns in RCCs, we speculate that it is highly likely for CPD-induced *TP53* mutations to be involved in RCC carcinogenesis, or at least in RCC tumor progression.

### 4.2. Study Limitations and Validation Issues

Notwithstanding the experimental evidence reviewed above regarding murine model CPD-driven *TP53* mutagenesis in cutaneous tumors, the current study design only allows for speculative observations regarding the pathogenic significance of pre-mutagenic CPDs in RCC carcinogenesis. From this viewpoint, the main limitation of the current investigation is conceptual. For the purpose of mere detection of TDs in the RCC samples evaluated, we decided to use IHC, an accessible yet semi-quantitative investigation at best. TD-targeted immunostaining only offers a static, singular, morphologic snapshot of the TD expression patterns within a pleomorphic, dynamic, and extremely complex biological system of molecular events, i.e., the RCC microenvironment.

Beyond its innate limitations, IHC also poses technical and interpretative challenges. For all markers, sensitivity/specificity will be dependent on multiple factors: clone/detection technique used, size/quality of specimen analyzed, and renal tumor grade. Necrosis or small tumor specimens, such as percutaneous biopsy cores or fine-needle aspiration samples, may affect the sensitivity of focal staining markers, while differentiated, low-grade RCC areas will provide improved immunoreactivity. Conversely, the variability among available studies regarding IHC technique and antibody variants (monoclonal/polyclonal, specific clone) hinders comparisons and precludes definitive conclusions [2]. Integration and standardization of both quantitative staining parameters and marker expression pattern analysis in IHC reports are essential [4].

To address these issues, in the current study, we used a fully automated IHC staining technique (Bond Max autostainer, Leica Biosystems) and a well-validated clone used in a variety of previous TD-targeted immunostaining investigations—mostly in human skin [82,83,84]—but also renal tissue, albeit after in situ hybridization with mRNA-targeted T-T dimerized synthetic oligonucleotides [85]. To facilitate reproducibility, we provide, to the best of our ability, a standardized and transferable protocol for reporting IHC results using quantification scores. Even so, we must acknowledge the lingering internal validation issues posed by the currently reported IHC investigation’s protocols, both regarding staining technique, but also the result of quantification and interpretation.

Inherently, due to the novelty of our study’s design, our results are the first of their kind and lack any preexisting terms of comparison. The clone currently used for TD detection is the oldest commercially available IHC antibody specifically targeting PDs. Notably, this particular clone, KTM53, was designed to only target CPDs (non-discriminately) and does not react with 6–4 PPs. Thus, theoretically, KTM53 should demonstrate an improved specificity for TDs, i.e., the most frequently occurring type of CPD, as compared to other subsequent PD-targeted clones. Even so, regardless of the specific antibody clone used, the possibility of non-specific binding of TD-targeted antibodies to unrelated antigens, which merely resemble the intended target structurally, remains a pervasive, major investigational pitfall of IHC. Therefore, at least to a certain degree, the currently reported results may be distorted by false-positive TD-immunoreactions.

Furthermore, the currently employed positive control, i.e., untreated in vivo skin used to validate our TD-targeted immunostaining method, is not ideal, lacking standardization and overall predictability. Despite being known to sporadically demonstrate TD immunoreactivity, preferentially in the basal epidermal cellularity and deeper dermal structures, natively UVR-exposed, paraffin-embedded, healthy, in vivo skin samples will most often be negative for TDs. Unsurprisingly, to obtain the relevant TD-immunostaining evidence included herein, 10 distinct skin samples had to be stained and evaluated. As expected, although mostly negative, heterogeneous areas of consistent nuclear TD-immunoreactivity were encountered. The evidence obtained was considered sufficient to confirm the validity of the automated TD-targeted immunostaining method currently employed. Conversely, notwithstanding concurrent interpretative biases, the high overall prevalence and the consistently displayed, distinctive expression patterns and preferential distribution of the TD immunoreactivity reported among the currently evaluated RCC cohort are unlikely to be purely the result of methodological errors. Ideally, a standardized protocol for controlled, pre-sampling UVR-exposure, of positive control, in vivo skin specimens should be developed.

Lastly, even though the results reported in the current study are novel, they have not yet been thoroughly validated, neither by using other commercially available TD-targeted antibody clones (i.e., the H3 clone from GeneTEX and/or the ab10347 clone from Abcam) nor by additional molecular techniques, i.e., enzyme-linked immunosorbent assay (ELISA), Southern blot and/or quantitative real-time polymerase chain reaction (qRT-PCR). Even so, these early findings regarding the TD expression in RCCs/renal tissue cannot be disregarded as merely artefactual and irrelevant, especially when taking into account their potentially significant implications regarding both the pathogenesis of PDs, as well as RCC carcinogenesis. For this reason and in order to expedite the external validation process, we provide the current early IHC report, regarding novel TD expression in RCCs. To further bolster the previously defined speculative model regarding TD-driven RCC carcinogenesis, we aim to follow up the current investigation with complementary IHC staining of the TD-positive RCC specimens, targeting the p53 transcript.

Statistical analysis of the results is also provided, yet, due to the modest cohort of RCC samples evaluated, the statistically significant correlations found, i.e., RCC tumor tissue TD-positivity associates lower TNM stage at diagnosis and stromal/endothelial TD expression in RCC adjacent healthy renal tissue associates RCC lymphatic dissemination, are unreliable and definitely require further validation as well.

## 5. Conclusions

In conclusion, the current study represents the very first investigation focused on evaluating the expression of TDs in RCC tumor tissue and tumor-adjacent healthy renal tissue using a TD-targeted IHC monoclonal antibody, clone KTM53. Our results illustrate a novel and pronounced association between RCCs and a mutagenic genomic lesion of great biological significance, previously thought to occur exclusively during UVR exposure. Out of the 54 RCC specimens evaluated, 77.8% showed nuclear TD-expression in RCC tumor tissue and 37% in the tumor-adjacent healthy renal parenchyma. A comprehensive report regarding quantitative and qualitative TD-targeted nuclear immunostaining parameters was elaborated. We also report the identification of two main distribution models for TD expression within RCC tumor tissue. Further rigorous molecular analysis is required in order to fully comprehend/validate the biological significance of this newly documented expression of TDs in RCC.

## Figures and Tables

**Table 1:** Definitions for TD immunoreaction scores in RCC tumor tissue, i.e., the quantitative (SQ), qualitative (immunoreaction intensity) (SI) and overall (ST) scores.

Grade	SQ Definition	SI Definition	ST Definition (SQ + SI)
0	Negative for TD.	Negative for TD.	Negative for TD.
+1	Rare positive nuclei (1–10% of total tumor cells), in a 400× field.	Weak immunoreaction, visibly less intense than external control	Cannot occur.
+2	Positive nuclei in 11% to 25% of the total tumor cell population, in a 400× field.	Moderate immunoreaction, similar or lesser intensity to external control skin TD staining	1 + 1
+3	Positive nuclei in >26% of the total tumor cell population, in a 400× field.	Strong immunoreaction, visibly as or more intense than external control skin TD staining	1 + 2 or 2 + 1
+4			1 + 3 or 2 + 2 or 3 + 1
+5			2 + 3 or 3 + 2
+6			3 + 3

**Table 2:** Comparison of patient characteristics stratified by the presence of thymine dimer staining in the tumor tissue of evaluated RCC samples.

Variables	TD Positive Tumor Tissue (*n* = 42)	TD Negative Tumor Tissue (*n* = 12)	*p*-Value
Age, years (mean ± SD)	65.74 ± 7.201	67.50 ± 10.113	0.499
**Sex**			0.402
Men	26 (48.15%)	9 (16.65%)	
Women	16 (29.65%)	3 (5.55%)	
**Histology (HP)**			0.703
ccRCC	30 (55.55%)	9 (16.65%)	
pRCC	6 (11.1%)	2 (3.7%)	
chRCC	4 (7.4%)	0 (0%)	
svRCC	2 (3.7%)	1 (1.85%)	
**Local extension (pT)**			0.189
1A	15 (27.8%)	2 (3.7%)	
1B	12 (22.2%)	1 (1.85%)	
2A	7 (12.95%)	5 (9.25%)	
2B	4 (7.4%)	2 (3.7%)	
3A	4 (7.4%)	2 (3.7%)	
**Fuhrman score**			0.793
1	12 (22.2%)	3 (5.55%)	
2	19 (35.2%)	7 (12.95%)	
3	8 (14.8%)	1 (1.85%)	
4	3 (5.55%)	1 (1.85%)	
**Lymph nodes (pN)**			0.665
Yes	5 (9.25%)	2 (3.7%)	
No	37 (68.5%)	10 (18.5%)	
**Distant metastasis**			0.634
Yes	2 (3.7%)	1 (1.85%)	
No	40 (74%)	11 (20.35%)	
**Stage at diagnosis**			0.055
1	29 (53.7%)	3 (5.55%)	
2	7 (12.95%)	5 (9.25%)	
3	4 (7.4%)	3 (5.55%)	
4	2 (3.7%)	1 (1.85%)	
**TD positive adjacent healthy** **renal tissue**			0.098
Yes	18 (33.35%)	2 (3.7%)	
No	24 (44.4%)	10 (18.5%)	

Data reported as *n* (frequency) and calculated using Pearson’s Chi-square test and Fisher’s exact test, unless specified differently; TD, Thymine Dimer; ccRCC, Clear Cell Renal Cell Carcinoma; pRCC, papillary Renal Cell Carcinoma; chRCC, chromophobe Renal Cell Carcinoma; chRCC, sarcomatoid variant Renal Cell Carcinoma.

**Table 3:** IHC staining characteristics in tumor tissue TD-positive RCCs.

Variables	TD-Positive Tumor Tissue (*n* = 42)
**Quantitative score**	
1	9 (21.4%)
2	18 (42.8%)
3	15 (35.7%)
**Intensity score**	
1	16 (38.1%)
2	19 (45.2%)
3	7 (16.7%)
**Overall expression score**	
2	5 (11.9%)
3	11 (26.2%)
4	12 (28.6%)
5	10 (23.8%)
6	4 (9.5%)

Data reported as *n* (frequency) and calculated using Chi-square test and Fisher’s exact unless specified differently; TD—Thymine Dimers.

**Table 4:** Correlation analysis of the studied variables.

		Age	Sex	RS	cN	Stage	FG	HKE	HSE	TD+/−	SQ	SI	ST
**Age**	*Rho*	**1**	−0.234	−0.420 **	−0.149	−0.159	−0.061	−0.235	0.101	−0.136	0.007	−0.114	−0.040
	*p−value*	-	0.088	0.002	0.283	0.251	0.662	0.087	0.465	0.327	0.961	0.410	0.773
**Sex**	*Rho*	−0.234	**1**	0.242	0.177	0.104	0.046	0.238	0.186	0.114	−0.043	0.248	0.100
	*p−value*	0.088	-	0.078	0.199	0.453	0.744	0.083	0.177	0.412	0.759	0.071	0.471
**RS**	*Rho*	−0.420 **	0.242	**1**	0.420 **	0.261	0.159	0.245	−0.084	0.036	−0.041	−0.115	−0.102
	*p−value*	0.002	0.078	-	0.002	0.056	0.250	0.074	0.546	0.794	0.769	0.407	0.461
**cN**	*Rho*	−0.149	0.177	0.420 **	**1**	0.615 **	0.356 **	0.161	0.356 **	−0.059	−0.088	0.022	−0.031
	*p−value*	0.283	0.199	0.002	-	0.000	0.008	0.246	0.008	0.672	0.526	0.874	0.826
**Stage**	*Rho*	0.251	0.104	0.261	0.615 **	**1**	0.295 *	0.099	0.205	−0.353 **	−0.212	−0.195	−0.170
	*p−value*	0.540	0.453	0.056	0.000	-	0.031	0.476	0.138	0.009	0.123	0.157	0.220
**FG**	*Rho*	−0.061	0.046	0.159	0.356 **	0.295 *	**1**	0.111	0.176	0.025	0.064	0.109	0.068
	*p−value*	0.662	0.744	0.250	0.008	0.031	-	0.423	0.204	0.860	0.644	0.433	0.625
**HKE**	*Rho*	−0.235	0.238	0.245	0.161	0.099	0.111	**1**	0.179	0.225	0.341 *	0.353 **	0.394 **
	*p−value*	0.087	0.083	0.074	0.246	0.476	0.423	-	0.195	0.101	0.012	0.009	0.003
**HSE**	*Rho*	0.101	0.186	−0.084	0.356 **	0.205	0.176	0.179	**1**	0.073	0.179	0.216	0.225
	*p−value*	0.465	0.177	0.546	0.008	0.138	0.204	0.195	-	0.598	0.196	0.116	0.103
**TD+/−**	*Rho*	−0.136	0.114	0.036	−0.059	−0.353 **	0.025	0.225	0.073	**1**	0.748 **	0.752 **	0.734 **
	*p−value*	0.327	0.412	0.794	0.672	0.009	0.860	0.101	0.598	-	0.000	0.000	0.000
**SQ**	*Rho*	0.007	−0.043	−0.041	−0.088	−0.212	0.064	0.341 *	0.179	0.748 **	**1**	0.692 **	0.917 **
	*p−value*	0.961	0.759	0.769	0.526	0.123	0.644	0.012	0.196	0.000	-	0.000	0.000
**SI**	*Rho*	−0.114	0.248	−0.115	0.022	−0.195	0.109	0.353 **	0.216	0.752 **	0.692 **	**1**	0.905 **
	*p−value*	0.410	0.071	0.407	0.874	0.157	0.433	0.009	0.116	0.000	0.000	-	0.000
**ST**	*Rho*	−0.040	0.100	−0.102	−0.031	−0.170	0.112	0.394 **	0.225	0.734 **	0.917 **	0.905 **	**1**
	*p−value*	0.773	0.471	0.461	0.826	0.220	0.421	0.003	0.103	0.000	0.000	0.000	-

** Correlation is significant at the 0.01 level (2-tailed); * Correlation is significant at the 0.05 level (2-tailed); RS, RCC subtype; FG, Fuhrman grade; HKE, tumor adjacent healthy renal tissue TD expression; HSE, healthy renal tissue stromal/endothelial TD expression; TD+/−, Thymine Dimer expression in RCC tumor tissue; SQ, quantitative score for TD+/−; SI, qualitative score for TD+/−; ST, cumulative expression score for TD+/−.

## Data Availability

Our data are available at https://data.mendeley.com/datasets/3pmnxwhgxk/1 (accessed on 29 July 2022).
